# How Old Do You Feel? The Role of Age Discrimination and Biological Aging in Subjective Age

**DOI:** 10.1371/journal.pone.0119293

**Published:** 2015-03-04

**Authors:** Yannick Stephan, Angelina R. Sutin, Antonio Terracciano

**Affiliations:** 1 University of Montpellier, Montpellier, France; 2 College of Medicine, Florida State University, Tallahassee, Florida, United States of America; Cardiff University, UNITED KINGDOM

## Abstract

Subjective age, or how young or old individuals experience themselves to be relative to their chronological age, is a crucial construct in gerontology. Subjective age is a significant predictor of important health outcomes, but little is known about the criteria by which individuals' subjectively evaluate their age. To identify psychosocial and biomedical factors linked to the subjective evaluation of age, this study examined whether perceived age discrimination and markers of biological aging are associated with subjective age. Participants were 4776 adults (*M*_age_ = 68) from the 2008 and 2010 waves of the Health and Retirement Study (HRS) who completed measures of subjective age, age discrimination, demographic variables, self-rated health and depression, and had physical health measures, including peak expiratory flow, grip strength, waist circumference, systolic and diastolic blood pressure. Telomere length was available for a subset of participants in the 2008 wave (n = 2214). Regression analysis indicated that perceived age discrimination, lower peak expiratory flow, lower grip strength, and higher waist circumference were associated with an older subjective age, controlling for sociodemographic factors, self-rated health, and depression. In contrast, blood pressure and telomere length were not related to subjective age. These findings are consistent with the hypothesis that how old a person feels depends in part on psychosocial and biomedical factors, including the experiences of ageism and perceptible indices of fitness and biological age.

## Introduction

Subjective age refers to how young or old individuals experience themselves to be, relative to their chronological age. A growing body of research indicates that a younger subjective age is associated with a range of positive outcomes in old age, including higher psychological well-being [1–3], better physical and cognitive functioning [4,5], and longevity [6,7]. These associations persist even when controlling for chronological age, demographic and health-related variables [4–6]. Many of these studies test subjective age as a predictor of important outcomes at older ages; less research has addressed the variables associated with how individuals' subjectively evaluate their age.

Two non-mutually exclusive theoretical perspectives have developed in parallel on the predictors of subjective age. From a social psychological perspective, the age an individual feels is associated with social and environmental cues, particularly information about aging [5, 8, 9]. From a biomedical perspective, how old or young an individual feels is sensitive to information about one's physical health and functioning [10–12]. To date, most research has focused on either psychosocial or biomedical factors, with little research that considers these different perspectives simultaneously. Drawing upon these theoretical accounts, the present study examines the links between subjective age and both psychosocial and biomedical indicators. From a psychosocial perspective, we examine whether subjective age is associated with the perceived experience of age discrimination. From a biomedical perspective, we examine whether subjective age is associated with a variety of clinical and laboratory assessments that account for individuals’ physical fitness and health condition.

The extent to which individuals feel discriminated against because of their age is a significant social experience that may contribute to how old or young they feel. With advancing age, individuals are increasingly exposed to negative stereotypes of aging, which may translate into social devaluation, unfair treatment, and age discrimination. Discrimination reflects rejection and social exclusion, and is considered a significant social stressor that has harmful effects for physical and mental health [13]. There are reasons to expect that subjective age would be sensitive to perceived age discrimination. Experimental evidence suggests that subjective age changes in response to age-related social cues and information [5, 8, 14]. For example, older adults feel older after being exposed to negative aging stereotypes [8]. This pattern suggests an assimilation effect, in which stereotypical negative information about old age is integrated into individuals' self-perceptions [9], resulting in an older subjective age [8]. These findings thus support the hypothesis that when exposed to age discrimination, individuals may assimilate the negative age-related information conveyed by such discriminatory experiences, which increases the likelihood that they will feel older. Existing research, however, has focused on the effects of activating aging stereotypes, not the association between perceived age discrimination and subjective age.

Compared to the literature on the psychosocial correlates of subjective age, less research has addressed the role of biological markers in subjective age. Most studies are based on either self-report measures of health or the number of conditions and illness diagnosed by a physician [6, 10]. Few studies have included biomarkers of health and fitness assessments, such as staff-assessed anthropometric assessments or laboratory-based assays. Given the literature linking subjective age to morbidity and mortality, it is likely that feeling subjectively younger is associated with slower physiological aging, better physical fitness, and better health. This hypothesis is based on evidence that broad physiological processes and afferent biological messages from different bodily systems are integrated into self-assessments through interoception [15, 16]. Interoception refers to the ability to detect stimuli and changes in bodily systems through a complex network of physiologic receptors attuned to the body conditions, including pain, muscle tension, itch, and breath [17, 18]. Even bodily irregularities and dysfunctions too minor to be defined as medical conditions or conceptualized as illness can be sensed and integrated into self-assessments [16]. As such, subjective age may be related to clinical and physical performance measures that are important for the health and functioning of the individuals, but are not necessarily diseases.

In this study, we focus on measures of muscle strength, aerobic capacity, metabolic, cardiovascular, and cellular aging. These measures should be relevant to subjective age because with aging there are significant declines in strength, cardiovascular fitness, and energy level [19, 20], which all play an important role for individuals’ functioning and lifestyle. Indeed, better respiratory and muscular functions, and lower adiposity relate to less limitations in daily activities [21–23] which may be reflected in a younger subjective age. With age there is also shortening of telomeres (nucleoprotein structures located at the ends of eukaryotic chromosomes that protect chromosome integrity) [24] a major marker of cellular senescence [25]. Longer telomeres are associated with a lower rate of age related disease (e.g., cancer), and higher life expectancy [26]. Because of such links with cellular aging, diseases, and longevity, it is likely that longer telomere length is associated with a younger subjective age. Specifically, we will test whether biomarkers such as systolic and diastolic blood pressure, peak expiratory flow, grip strength, waist circumference, and telomere length are associated with subjective age. Systolic and diastolic blood pressure are indicators of cardiovascular functioning, peak expiratory flow reflects the functioning of the respiratory system, grip strength indexes muscle mass and strength, waist circumference is a marker of central adiposity and metabolic dysregulation, and telomere length is a marker of cellular aging.

To summarize, this study aims to identify social and biological correlates of subjective age. In line with evidence for the psychological consequences of discrimination [13] and the effect of aging stereotypes on subjective age [8], we hypothesize that perceived age discrimination will be related to an older subjective age. In line with studies on biological age [19, 20], we expect that even after accounting for chronological age and other relevant sociodemographic factors, stronger grip strength, greater peak expiratory flow, lower waist circumference and blood pressure, and longer telomeres will be associated with a younger subjective age.

## Materials and Methods

### Participants

Participants were drawn from the Health and Retirement Study (HRS), a nationally representative longitudinal study of Americans ages 50 and older sponsored by the National Institute of Aging (grant number NIA U01AG009740) and conducted by the University of Michigan. HRS participants are re-interviewed every two years. Starting in 2006, HRS implemented an enhanced face-to-face interview that included the biomarker measurements and a psychosocial questionnaire that was left to participants at the end of the interview to complete at home and returned by mail. Subjective age was first assessed in this psychosocial questionnaire in 2008. Half of the HRS participants completed the enhanced interview that included subjective age, age discrimination and the biological measures in 2008 and the other half completed these assessments in 2010. Data from both waves were pooled, and only participants who had available data on all measures of interest were included in the present study. The final sample was composed of 4776 participants (57% women), who were, on average 68.50 (*SD* = 9.50, range: 50–100 years) years old, and had an average of 12.92 (*SD* = 3.03) years of education. This sample was 83% white, 14% African American, and 3% from other races. In addition, 9% of participants were Hispanic. Telomere length was available for a subsample of the 2008 respondents (N = 2255) within the final sample.

### Ethics Statement

The Health and Retirement Study is conducted under Institutional Review Board approval by the relevant committees at the University of Michigan and the National Institute on Aging, the primary sponsor of HRS. Prior to describing the individual measures to participants in the enhanced face-to-face interview, a consent form was administered by the interviewer, where the participants were asked to read and sign the form. Participants who did not sign the consent form were not asked to complete the measures. Separate consent forms were administered for the saliva and blood samples. Each form was introduced just prior to the measure(s) that it covered. After obtaining consent for a given component, the interviewer described the procedures to the respondent and demonstrated how the measure was conducted. This consent procedure was approved by the ethic committee.

### Measures

#### Subjective age

Subjective age was assessed by asking participants to specify, in years, how old they felt. Proportional discrepancy scores were calculated by subtracting participants’ felt age from their chronological age, and these difference scores were divided by chronological age [5, 14, 27]. A positive value indicated a youthful subjective age, and a negative value indicated an older subjective age. For example, a participant who scored +0.20 felt 20% younger, whereas a participant who scored -0.20 felt 20% older than their actual age. In line with previous studies [4, 28], responses three standard deviations above or below the mean were considered outliers, leading to the exclusion of 75 participants from the analysis.

#### Age discrimination

Participants were first asked whether they have experienced everyday discrimination, such as being treated with less respect than other people, or receiving poorer service than other people [29]. They were then asked whether they thought these discriminatory experiences were due to a number of personal characteristics, including age, using a single item [30]: “If any of the above have happened to you, what do you think were the reasons why these experiences happened to you? (Mark all that apply.)" Participants who reported being discriminated against because of their age were coded as 1 and those who did not report age discrimination were coded as 0. Although single-item measures are not ideal, they have been used successfully to examine the effect of age discrimination on health-related outcomes [31].

#### Blood pressure

From a sitting position, three blood pressure readings, 45 seconds apart, were taken using an Omron HEM-780 Intellisense Automated Blood Pressure Monitor on the respondent's left arm. Systolic and diastolic blood pressure were recorded and averaged across the three measurements (mmHg).

#### Peak Flow

The peak expiratory flow test consisted of three measurements taken 30 seconds apart using a Mini-Wright peak flow meter (Clement Clarke International Ltd., Harlow, United Kingdom). The maximum volume expired (liters per minute) of the three attempts was used in the analyses.

#### Grip strength

Hand grip strength was measured in kilograms using a Smedley spring type hand dynamometer. The measure was conducted with the respondent standing and holding the dynamometer at a 90 degree angle. Two measurements were taken on each hand alternating between the left and right hand. The best score of the two attempts for the dominant hand was used in the analyses.

#### Waist circumference

Respondents were asked to stand up and remove any bulky clothing, point to their navel and place a tape measure around their waist at the level of their navel. The interviewer checked to be sure that the tape measure was horizontal around the waist and snug but not tight. Waist circumference in inches was recorded and used in the present study.

#### Telomere length

Telomere length was obtained from a subsample of 2008 HRS respondents who consented and provided a saliva sample using an Oragene Collection Kit during the 2008 interview wave. Average telomere length was assayed using quantitative PCR (qPCR) [32] by comparing telomere sequence copy number in each participant’s sample (T) to a single-copy gene copy number (S). The resulting T/S ratio is proportional to mean telomere length [32]. The mean T/S ratio in this study (range: 0.23–19.31) was similar to prior reports on older adults [33].

#### Covariates

Age (in years), sex (coded as 1 for men and 0 for women), and educational level (in years) were included as covariates. Age squared was also included to account for a potential non-linear association between chronological age and subjective age. Based upon existing studies on race and ethnic disparities in health and cognition [34, 35], race was controlled and coded as 1 for white and 0 for black and others, and ethnicity was coded as 1 for Hispanic and 0 for non-Hispanic. Given that self-rated health and depressive symptoms are important predictors of subjective age [12, 36], they were also included as covariates. Self-rated health was assessed using a single item that asked participants to report whether their health was excellent, very good, good, fair, or poor. We reversed the original scale so that excellent health was the highest score (5) and poor health was the lowest (1). Depressive symptoms were measured using an 8-item version of the Centers for Epidemiologic Research Depression (CES-D) [37]. Participants were asked to report whether they had experienced (yes/no) eight specific symptoms for much of the past week. The total number of endorsed symptoms was summed to create a total depressive symptom score ranging from 0 to 8 (α = .81).

### Data Analysis

A multiple regression analysis was conducted with subjective age as the outcome and age discrimination and the biomarkers as predictors; sociodemographic factors (age, age squared, sex, education, race and ethnicity), as well as self-rated health and depressive symptoms were included as covariates. Collinearity diagnostics indicated that there were no problems with multicollinearity.

## Results

Table 1 shows the descriptive statistics for all study variables. We first conducted partial correlations between subjective age and discrimination and the biological markers of health, controlling for age, age squared, sex, education, race and ethnicity. Age discrimination (*r* = -0.07, *p* <.001), peak expiratory flow (*r* = 0.12, *p* <.001), grip strength (*r* = 0.12, *p* <.001) and waist circumference (*r* = -0.15, *p* <.001) were related to subjective age in the hypothesized direction; systolic blood pressure (*r* = 0.01, *p* = .42), diastolic blood pressure (*r* = 0.02, *p* = .28), and telomere length (*r* = 0.02, *p* = .27) were not associated with subjective age.

**Table 1:** Sample Characteristics (N = 4701).

Variables	M/%	SD
Sex (% female)	57%	-
Age (years)	68.54	9.50
Education	12.93	3.03
Race (% white)	83%	-
Ethnicity (% hispanic)	9%	-
Self-rated health	3.16	1.04
Depressive symptoms	1.46	1.98
Age Discrimination (% discriminated)	56%	-
Systolic blood pressure (mmHg)	130.77	20.03
Diastolic blood pressure (mmHg)	78.96	11.42
Peak expiratory flow (liters/mn)	367.45	133.11
Grip strength (kg)	30.98	11.00
Waist circumference (inches)	40.06	6.06
Telomere length (T/S Ratio)^a^	1.39	0.74
Subjective age ^b^	0.15	0.15

*Note*.

^a^ *N* = 2214.

^b^ Higher values represent younger subjective age.

Seventy-five participants who were outliers on subjective age were excluded from the analysis.

**Fig 1:**
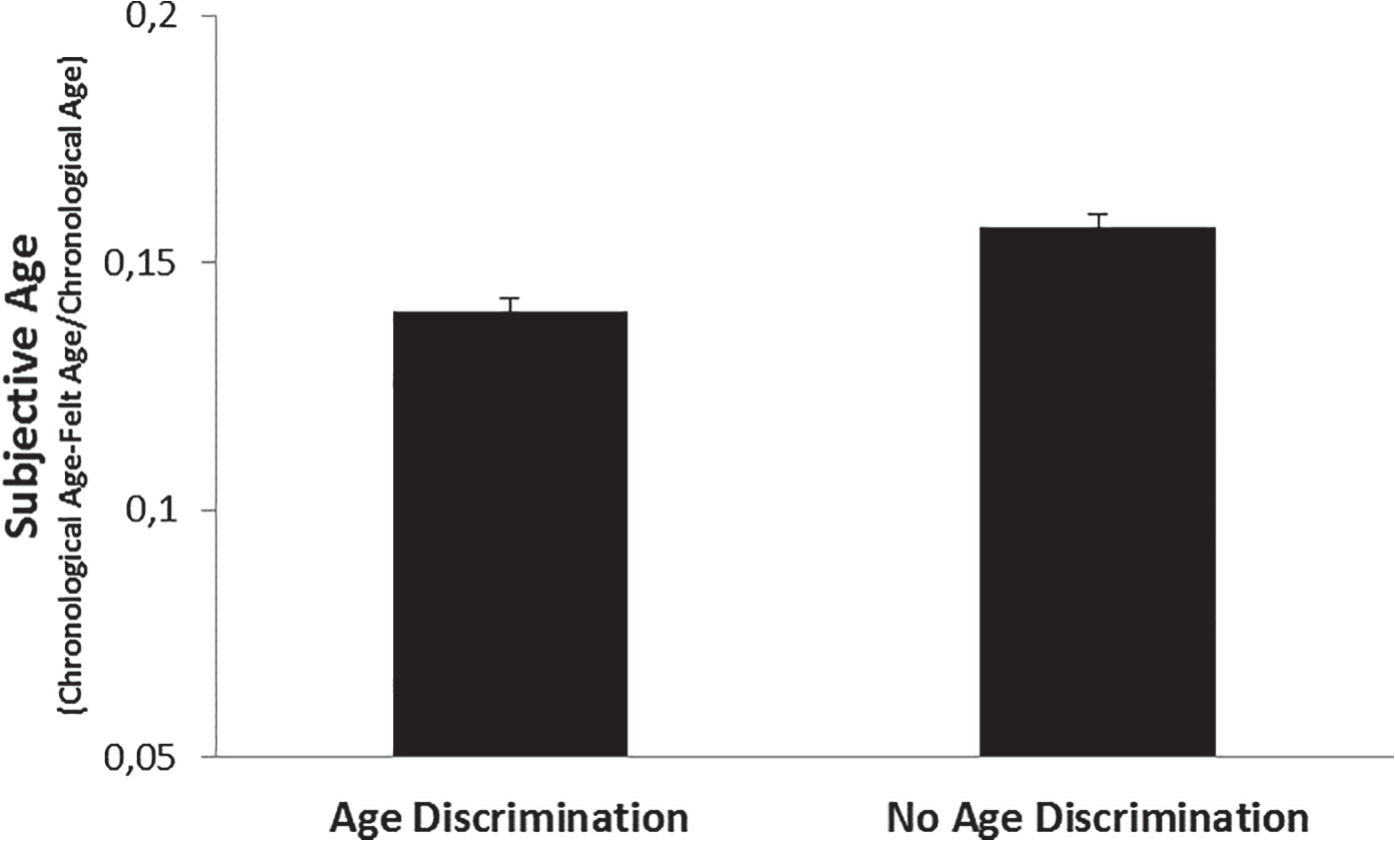
Subjective Age as a Function of Age Discrimination. Higher Values on the y-Axis represent Younger Subjective Age. Values are Adjusted for Covariates and Biomarkers

As expected, the regression analysis indicated that age discrimination was negatively related to subjective age, controlling for the demographic variables, self-rated health, and depressive symptoms (Table 2). Individuals exposed to age discrimination felt approximately 2% older than those who did not report such experience (mean subjective age discrepancy score: 0.14 vs 0.16) (Fig. 1). For example, a 68-year-old individual who experienced age discrimination felt around one year older than a 68-year-old individual without such experiences.

**Table 2:** Summary of Regression Analysis Predicting Subjective Age from Age Discrimination, Biomarkers, and Covariates.

Variables	B	SEB	β
Sex	-0.030	0.006	-0.10***
Age	0.001	0.000	0.09***
Age Squared	-0.000	0.000	-0.06***
Education	0.002	0.001	0.04**
Race	-0.028	0.005	-0.07***
Ethnicity	0.035	0.008	0.06***
Self-rated health	0.036	0.002	0.24***
Depressive symptoms	-0.006	0.001	-0.09***
Age Discrimination	-0.016	0.004	-0.05***
Systolic blood pressure	0.000	0.000	0.01
Diastolic blood pressure	-0.000	0.000	-0.002
Peak expiratory flow	0.000	0.000	0.06***
Grip strength	0.001	0.000	0.09***
Waist circumference	-0.003	0.000	-0.10***

*Note*. *N* = 4701.

Adjusted *R* ^*2*^ = .141.

*F*(14, 4686) = 56.39.

*p*<.001.

* *p*<.05.

** *p*<.01.

*** *p*<.001.

**Fig 2:**
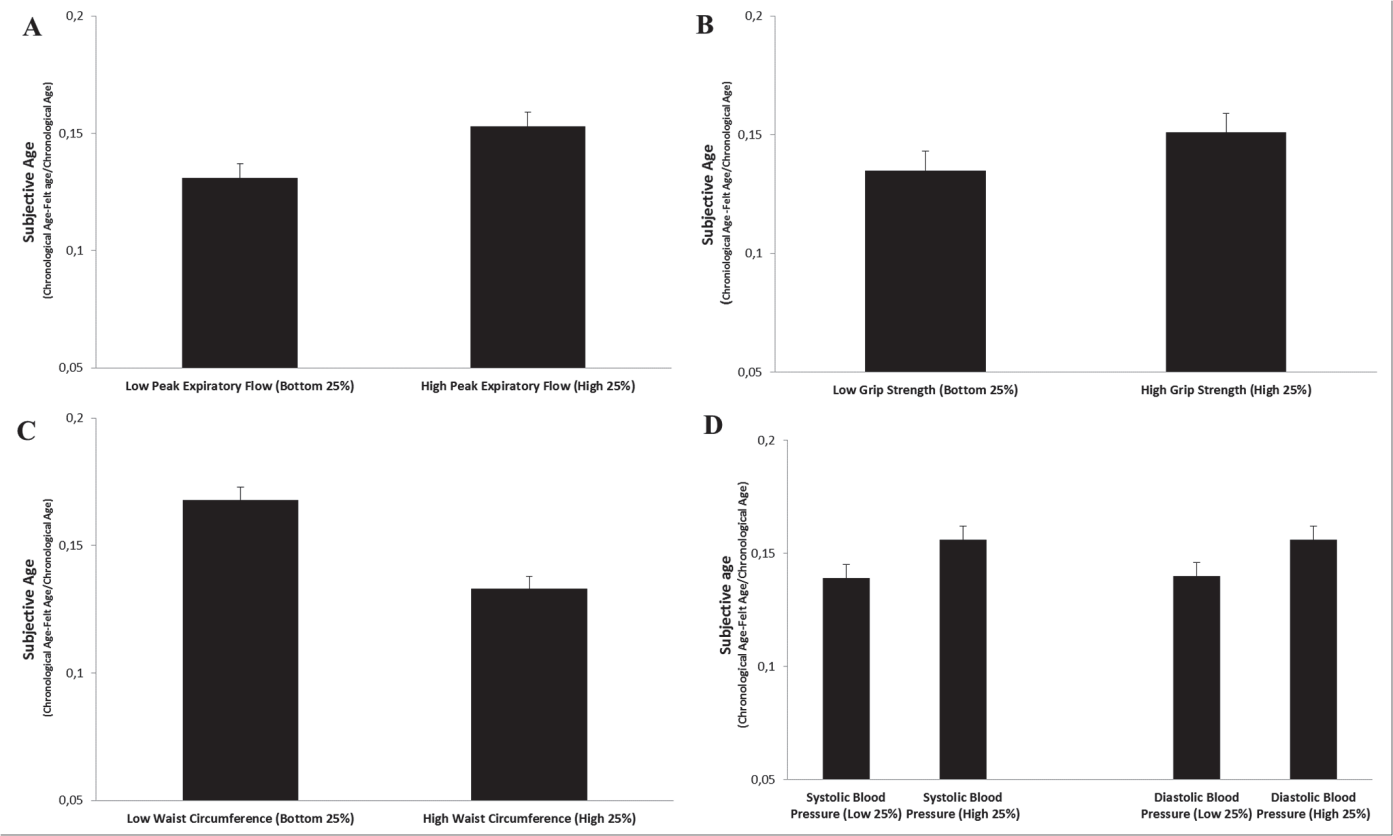
Subjective Age for Low (Bottom 25%) and High (High 25%) Peak Expiratory Flow (Panel A), Grip Strength (Panel B), Waist Circumference (Panel C), and Systolic Blood Pressure and Diastolic Blood Pressure (Panel D). Higher Values on the y-Axis represent Younger Subjective Age. Values are Adjusted for Covariates and Age Discrimination.

Consistent with our hypothesis, the regression also revealed that higher peak expiratory flow and grip strength were both associated with a younger subjective age, while higher waist circumference was related to an older subjective age, independent of the covariates (Table 2). There was no association between blood pressure and subjective age. Fig. 2 presents the subjective ages of individuals in the top and the bottom 25% of the distribution of peak expiratory flow, grip strength, waist circumference, and systolic and diastolic blood pressure, adjusted for the covariates. Individuals in the top quartile of grip strength and peak flow felt around 2% younger, than those in the bottom quartile (mean subjective age discrepancy score: 0.15 vs 0.13 for both grip strength and peak flow), whereas participants in the bottom quartile of waist circumference felt about 4% younger than those in the top quartile (0.17 vs 0.13). Compared to the top quartile, a 68-year-old individual in the bottom quartile of waist circumference reported feeling almost 3 years younger. The effect size for biomarkers were comparable or stronger than the effect of age discrimination. In addition, the effect size for age discrimination and the biomarkers were generally stronger or comparable to those of the demographic factors.

Finally, we conducted an additional analysis on the subsample of participants from the 2008 wave that had telomere length available. Telomere length was unrelated to subjective age in the regression that controlled for the demographic factors, depression, self-rated health, age discrimination and the biomarkers (*β* = .02, *p* = .42). The overall pattern of associations between age discrimination/biomarkers and subjective age was the same without the covariates included in the model and with adjusting for both blood pressure medication and height. Additional analysis revealed no sex differences in the overall pattern of relations. Analysis of the moderating role of age revealed only that the negative association between waist circumference and subjective age was stronger among relatively younger compared to relatively older participants in the sample (*β*_*i*nteraction_ = .03, *p*< .05).

## Discussion

In a large sample of adults aged 50 and older, we found support for our hypothesis that age discrimination and perceptible markers of biological aging contribute to subjective age, even after accounting for demographic factors, self-rated health and depressive symptoms. Consistent with our hypothesis, perceived age discrimination was related to an older subjective age, whereas higher peak expiratory flow, grip strength, and lower waist circumference were associated with a younger subjective age. Neither blood pressure nor telomere length was associated with subjective age.

The present study provides evidence for an association between perceived age discrimination and subjective age. It complements prior reports on the threatening role of exposure to negative aging stereotypes for individuals' subjective age [8] and further implicates negative social cues and experiences about one's age for how old the individual feels. Individuals exposed to age-related discriminatory experiences may be more likely to integrate negative views of aging into their own self-views, resulting in an older subjective age compared to those without such experiences. In addition, perceived discrimination is a significant social stressor that has negative consequences for physical and mental health [13, 31, 38] that may lead individuals to feel older.

The most novel finding of this study was the association between subjective age and biological aging, indexed by commonly assessed biomarkers. Specifically, markers of better pulmonary and muscular function and lower central adiposity were all independently related to a younger subjective age. These findings provide new evidence for the biological underpinnings of subjective age. Building upon existing research on interoception [17, 39], it is likely that when rating their felt age, individuals may be sensitive to the physiologic condition of the body. There are very physical manifestations of the respiratory and muscular systems and body composition that translate biological messages into clear feelings and sensations. That is, individuals with less strength, limited lung function, and more adiposity experience more functional limitations and difficulties in performing activities of daily living [21–23]. These difficulties may generate sensations of pain, fatigue, and breathlessness that lead to an overall feeling of being older. In contrast, better muscular and pulmonary functioning, as well as lower adiposity facilitates a physically active lifestyle [40–42], which is beneficial for a younger subjective age. Individuals with this biological aging profile may feel younger because they may experience fewer negative sensations and more positive feelings when making physical efforts. In contrast, the null effect for blood pressure and telomere length is consistent with the notion that people have limited awareness of their blood pressure and cellular aging. Indeed, elevated blood pressure is often referred to as the “silent killer” [43]. As such, with limited perception of physical symptoms that their blood pressure is abnormal, individuals may not incorporate blood pressure into their evaluation of how old they feel. In the same vein, the cellular senescence associated with shortening of telomeres is unlikely to be directly accessible to individuals. Therefore, these findings suggest that subjective age is more sensitive to the biological aging of critical body systems leading to physical symptoms that have implications for everyday functioning than to purely cellular aging or biomarkers without such physical signs.

The present study suggests that subjective age integrates both social and biological cues about aging and thus adds to prior theoretical accounts that consider social psychological and health-related factors as separate sources of information for age ratings. Taking into account the role of self-rated health and depression for important age-related outcomes, subjective age can be understood as a condensed summary of information about processes that are involved in cognitive and physical functioning. Indeed, lower peak expiratory flow and grip strength, higher waist circumference, and discriminatory experiences predict lower cognitive performance [20, 44, 45], and mortality risk [38, 46–48]. This evidence suggests that subjective age may contribute to health and cognition in old age because it reflects social and biological factors relevant to such outcomes. From a clinical standpoint, subjective age assessment may be a useful tool to identify individuals at increased risk for adverse outcomes in old age, and who may benefit from early intervention to alleviate psychological, cognitive and health-related deficits. Experimental evidence indicates that subjective age can be partly changed [5]. As such, subjective age might be a promising target for interventions to improve psychological and physical functioning [5].

The present research had several strengths, including a large sample and a comprehensive, simultaneous assessment of factors potentially associated with subjective age, ranging from sociodemographic to biological. Despite its strengths, this study has several limitations that should be considered. First, the observational study design limits our ability to determine causal associations among the variables under consideration. Although we focused on subjective age as the dependent variable, it is likely that subjective age could also contribute to biological functioning and the perception of age discrimination. Indeed, experimental evidence indicates that the social manipulation of subjective age is associated with changes in physical functioning [5]. Thus, subjective age may reflect psychological and biomedical conditions, and at same time might have pervasive influences on psychosocial and physical functioning. Second, the modest size of the associations reported in the present study suggests that other factors contribute to subjective age. Indeed, subjective age is likely to have a complex etiology, with many psychological, social, cultural, and biological antecedents. Other predictors need to be considered, such as personality traits, health behaviors, activities of daily living, and life events [12, 49]. Third, future research should test the pathways through which discrimination and biological age contribute to subjective age. Finally, although this research considered commonly used markers of biological aging, further studies may include additional important biomarkers, such as those of the immune and central nervous systems.

## Conclusion

The present study provides empirical evidence that advances our theoretical understanding of the role of nature and nurture in the subjective evaluations of age. Beyond well-established predictors, how old or young individuals feel is related to social and biological cues about aging. Therefore, it paves the way for future researchers interested in identifying the range of social and biological factors associated with subjective age that may ultimately explain the implications of this construct for a range of crucial age-related outcomes.
